# Microarrays

**DOI:** 10.1111/j.1467-7687.2007.00558.x

**Published:** 2007-01

**Authors:** Robert Plomin, Leonard C Schalkwyk

**Affiliations:** Social, Genetic and Developmental Psychiatry, Institute of PsychiatryLondon, UK

## Abstract

Microarrays are revolutionizing genetics by making it possible to genotype hundreds of thousands of DNA markers and to assess the expression (RNA transcripts) of all of the genes in the genome. Microarrays are slides the size of a postage stamp that contain millions of DNA sequences to which single-stranded DNA or RNA can hybridize. This miniaturization requires little DNA or RNA and makes the method fast and inexpensive; multiple assays of each target make the method highly accurate. DNA microarrays with hundreds of thousands of DNA markers have made it possible to conduct systematic scans of the entire genome to identify genetic associations with complex disorders or dimensions likely to be influenced by many genes of small effect size. RNA microarrays can provide snapshots of gene expression across all of the genes in the genome at any time in any tissue, which has far-reaching applications such as structural and functional ‘genetic neuroimaging’ and providing a biological basis for understanding environmental influence.

## Introduction

The goal of behavioural genomics is to understand the developmental pathways between genes and behaviour, not just for a single gene and a single behaviour, but for the system that includes all genes (the genome) and all behaviours (the behavioural phenome) (Plomin, DeFries, Craig & McGuffin, 2003). The first step in these pathways between the genome and the phenome is the transcriptome – DNA throughout the genome that is expressed (transcribed from DNA to RNA). Every cell with a nucleus has the same genome but only a minority of the genes are expressed in quantities large enough to have an effect. Development can be viewed as the process by which the genome becomes differently expressed in different cells. Gene expression is a highly complex and tightly regulated process that governs development and also allows a cell to respond to the environment inside and outside the cell. Not just an ‘on/off’ switch for each gene, gene expression is a ‘volume control’ that increases or decreases the level of expression as needed.

Although it might seem like science fiction, it is now possible to study the entire genome (DNA) and the transcriptome (RNA), thanks to DNA microarrays that can assess sequence variation in all the genes in the genome and RNA microarrays that can assess expression levels of all the RNA transcripts in the genome. Microarrays detect DNA and RNA in the traditional way by taking advantage of the double-stranded nature of DNA and the binding (hybridization) of short single-stranded bits of DNA and RNA to their complements. The difference is that microarrays can do this for all the genes in the genome on a slide the size of a postage stamp. This miniaturization makes it is possible to assess the entire genome or the entire transcriptome quickly in a cost-effective manner using very little DNA or RNA. Arrays are also very accurate because miniaturization makes it possible to assess each target many times. Details about how microarrays work are available elsewhere (http://www.ncbi.nlm.nih.gov/About/primer/microarrays.html; http://www.bio.davidson.edu/courses/genomics/chip/chip.html).

## DNA

Working with DNA is much easier than RNA because DNA does not change across time or tissue. That is, DNA can be obtained just once at any time during development because the DNA sequence does not change during development or in response to the environment. In addition, DNA can be extracted from any cells that have a nucleus; most frequently used are white blood cells (red blood cells do not have nuclei) and cells from the inside of the cheek. DNA microarrays are currently available commercially that genotype 500,000 DNA markers (called single-nucleotide polymorphisms, SNPs) distributed throughout the genome (Affymetrix, 2004).

SNP microarrays have made it possible to conduct genome-wide association scans for complex traits and common disorders whose genetic influence is likely to be due to many genes of small effect, called quantitative trait loci (QTLs) (Hirschhorn & Daly, 2005). Such genome-wide association scans also raise new analytic problems (Carlson, Eberle, Kruglyak & Nickerson, 2004; Freimer & Sabatti, 2005; Thomas, Haile & Duggan, 2005; Wang, Barratt, Clayton & Todd, 2005). Even though each microarray is expensive and can be used only once, many DNA microarray studies are under way genotyping thousands of subjects in order to achieve the power needed to detect QTLs of small effect size. It is also possible to reduce the cost of DNA microarray studies dramatically by pooling DNA from many individuals in a group (such as cases or controls) and genotyping the pooled DNA on a single DNA microarray (Butcher, Meaburn, Liu, Hill, Al-Chalabi, Plomin, Schalkwyk & Craig, 2004; Meaburn, Butcher, Liu, Fernandes, Hansen, Al-Chalabi, Plomin, Craig & Schalkwyk, 2005; Meaburn, Butcher, Schalkwyk & Plomin, 2006). This method, which we call SNP Microarrays and Pooling (SNP-MaP), averages allele frequency estimates biologically for each group rather than obtaining allele frequencies for each individual in a group and then averaging them statistically across the group. We have applied the SNP-MaP approach to identify four SNPs associated with general cognitive ability (Butcher, Meaburn, Knight, Sham, Schalkwyk, Craig & Plomin, 2005).

Finding genes is expensive and difficult but using genes in research once the genes are identified is inexpensive and easy. We predict that disorder-specific DNA microarrays will be widely used in all the life sciences to genotype not just a few candidate genes but rather hundreds of SNPs associated with each disorder. With microarrays it would not matter if there were thousands of SNPs. For example, many SNPs in each gene might be needed to capture the genetic variation of the gene, and different sets of SNPs are likely to be associated with different developmental stages, with different aspects of the disorder, with different sexes, etc.

The unique strength of DNA associations is that these correlations are causal: DNA sequence variations do not change in response to behaviour or the environment. For this reason, DNA microarrays that contain as many SNPs as needed to account for the heritable variation in disorders can offer an early warning system to predict problems before they emerge in development, leading to hope for behavioural and environmental interventions that can prevent the disorders.

## RNA

In contrast to DNA itself, the transcription of DNA to RNA is very labile and specific to tissues and to cells within tissues because gene expression is responsive to the cellular and extra-cellular environment. Because RNA degrades quickly, gene expression can be indexed by the number of RNA transcripts in a particular tissue. RNA microarrays make it possible to take snapshots of profiles of gene expression throughout the genome for different groups (most notably, cases versus controls), at different times (for example, during development or before and after interventions) and in different tissues (for example, in different brain regions). There are scores of studies that have investigated mean differences in gene expression profiles between groups including cases versus controls for psychiatric disorders (Konradi, 2005), as well as many reports of changes in gene expression profiling, for example, in response to drugs (Yuferov, Nielsen, Butelman & Kreek, 2005). Although a review in 2004 of 5000 early gene expression studies found little evidence for reliability or validity (Miklos & Maleszka, 2004), dramatic improvements have been made during the past three years in relation to gene expression arrays and their analysis (e.g. Allison, Cui, Page & Sabripour, 2006; Cobb *et al.*, 2005). Another problem, the difficulty of making sense of the huge amount of data generated by RNA arrays, has also been ameliorated by the availability of databases and programmes that can be used to organize regulated genes according to their function and cellular location (Allison *et al.*, 2006; Konradi, 2005).

Gene expression profiling of the brain is like structural genetic neuroimaging in that it can create an atlas of localized patterns of gene expression throughout the brain. Because genetic neuroimaging requires brain tissue, its use in the human species is limited to post-mortem brains and tissue samples removed during surgery such as tumours (Yamasaki, Koyanagi, Fujii, Itoh, Barrero, Tamura, Yamaguchi-Kabata, Tanino, Takeda, Fukuchi, Miyazaki, Nomura, Sugano, Imanishi & Gojobori, 2005), which raise questions about lack of control concerning gene expression at the time of death (Konradi, 2005). For this reason, structural genetic neuroimaging research has primarily been investigated in fruit flies and mice rather than the human species.

Structural brain maps of gene expression are fundamental because genes can only function if they are expressed. The next goal is functional genetic neuroimaging – studying changes in gene expression in the brain across time, for example, during development or following interventions such as drugs or cognitive tasks. Such research on brain function needs to use animal models. For example, research on mice is under way that aims to create an atlas of profiles of gene expression throughout the brain during learning and memory tasks in the Genes to Cognition research consortium (Grant, 2003; http://www.genes2cognition.org).

Because of the practical and scientific limitations of using post-mortem brain tissue, RNA microarrays will be much more widely applicable to human research if easily available tissue such as blood can be used for gene expression profiling. Although the blood–brain barrier makes it unlikely that gene expression in the brain will be reflected by gene expression in the blood, many disorders directly involve blood such as infections (Feezor *et al.*, 2004) and many other disorders that involve the brain also involve blood (e.g. hormonal concomitants of anxiety). Moreover, some links between gene expression profiles in blood and brain are possible (Gladkevich, Kauffman & Korf, 2004; Nicholson, Unger, Mangalathu, Ojaniemi & Vernon, 2004; Pahl, 2005). Although gene expression profiling in the blood cannot be used to localize patterns of gene expression in the brain, blood could be used to address some important questions, most notably, gene expression profile differences as a function of development or interventions.

It should be emphasized that gene expression is a phenotype that changes in response to the environment. For this reason, RNA microarrays could lead to a paradigm shift in studying environmental influences on complex behavioural disorders by focusing on profiles of gene expression, the most fundamental mechanism of environmental influence. This approach could provide a biological foundation upon which to build an understanding of more complex levels of environmental analysis typically studied in behavioural research. It could also have far-reaching impact on translational research by providing biomarkers for differential diagnosis and providing a biological basis for monitoring environmental interventions such as drugs and other therapies. At the most general level, a functional focus on the transcriptome in terms of its effect on complex disorders will lead to research that integrates the disorder-relevant transcriptome with the genome, proteome, and eventually the brain.

## Non-coding RNA

DNA and RNA microarrays are greatly accelerating the already fast pace of discovery in molecular genetics. As mentioned earlier, DNA arrays have made possible genome-wide association scans for QTLs, and RNA arrays have facilitated genome-wide expression profiling. Moreover, DNA arrays and RNA arrays are likely to open up completely novel areas for research (Hoheisel, 2006). One example is that research using DNA and RNA microarrays is broadening our search for functional sequences, which is the focus of this concluding section.

The genes coding for proteins comprise only about 2% of the 3 billion nucleotide bases of the human genome. Microarray research has shown that much more of the genome is transcribed into RNA but *not* translated into amino acid sequences, called non-coding RNA (Mattick, 2004). Moreover, non-coding RNA is increasingly being shown to be functional, suggesting the need for a much broader view of the word ‘gene’ (Mattick, 2004). Evidence for the functionality of non-coding RNA comes from microarray research showing that about half of all transcription factor binding sites are in non-coding DNA regions, which indicates that non-coding RNA is regulated (Bertone, Stolc, Royce, Rozowsky, Urban, Zhu, Rinn, Tongprasit, Samanta, Weissman, Gerstein & Snyder, 2004; Cawley, Bekiranov, Ng, Kapranov, Sekinger, Kampa, Piccolboni, Sementchenko, Cheng, Williams, Wheeler, Wong, Drenkow, Yamanaka, Patel, Brubaker, Tammana, Helt, Struhl & Gingeras, 2004). Non-coding RNA is now known to regulate gene expression by a growing list of mechanisms such as RNA interference, gene silencing, and DNA demethylation (Costa, 2005).

This broader view of functional sequences is especially important because polymorphisms in such DNA transcribed into functional non-coding RNA are likely to create subtle genetic variation in the highly pleiotropic and polygenic systems that develop into complex traits and common disorders. In contrast, most rare and severe single-gene disorders are caused by mutations in coding DNA (OMIM Online Mendelian Inheritance in Man; OMIM, 2004). However, identifying functional sequences in non-coding DNA is a more difficult problem, and it is also possible that sequences responsible for the regulation of gene expression may evolve more rapidly than structural genes (Pang, Frith & Mattick, 2006). We predict that polymorphisms in non-coding DNA will prove to be an important source of QTLs responsible for the heritability of complex human traits.

For these reasons, we predict that non-coding DNA and its RNA will emerge as a major player in research on the genome and transcriptome and their links with the phenome. DNA microarrays are currently available commercially that can genotype hundreds of thousands of SNPs throughout the genome, thus capturing genetic effects mediated by non-coding as well as coding DNA. Non-coding DNA may be the answer to the question why initial research using DNA microarrays in genome-wide association scans points to associations with SNPs in non-coding DNA regions (e.g. Butcher *et al.*, 2005). Such associations may be examples of the functional effects of non-coding DNA. Non-coding DNA may also be the reason why linkage studies that consistently point to a chromosomal region (such as linkage to the short arm of chromosome 6 for reading disability) have had difficulty pinpointing the gene responsible for the linkage. The linkages might not be due to genes in the traditional sense of coding DNA; the culprits might be non-coding DNA. Although extant DNA microarrays include SNPs disproportionately in or near coding DNA, they will eventually include all polymorphisms in the genome, whether in non-coding or coding DNA.

Extant RNA arrays only assess coding RNA. Another step function in the pace of discovery will occur when RNA arrays are available that assess the entire transcriptome, whether coding or non-coding RNA. This major advance will happen soon because so-called tiling microarrays are now available that sample DNA from the entire genome and can be used to identify all transcribed sequences (Bertone, Stolc, Royce, Rozowsky, Urban, Zhu, Rinn, Tongprasit, Samanta, Weissman, Gerstein & Snyder, 2004; Bertone, Trifonov, Rozowsky, Schubert, Emanuelsson, Karro, Kao, Snyder & Gerstein, 2006; Washietl, Hofacker, Lukasser, Huttenhofer & Stadler, 2005). Such research has been facilitated by the commercial availability of standardized microarrays such as the Affymetrix GeneChip® Tiling Array Set 1.0R which provides 35-bp probe spacing of 45 million oligonucleotide probes which can be used for unbiased RNA transcript mapping.

## Conclusions

The impact of DNA and RNA microarrays on developmental science will be enormous. Developmental science will relate the genome and transcriptome to the behavioural phenome as it develops at all levels of analysis from proteins to the brain. For each phenotype, we can expect to identify different but overlapping sets of hundreds of relevant DNA polymorphisms and RNA transcripts at different developmental stages and in response to different experiences and different treatments. As such trait-specific, stage-specific and state-specific DNA and RNA microarrays become available commercially, we predict that they will be used routinely in developmental research including translational research that identifies new drug targets, leads to improved diagnoses including premorbid diagnoses, to personalized treatments, and especially to prevention of common developmental disorders.
